# Novel Application of Erector Spinae Plane Block to Interspinous Spacer Placement

**DOI:** 10.7759/cureus.11015

**Published:** 2020-10-18

**Authors:** David Hao, Vwaire Orhurhu, Joshua Hirsch, Zubin Irani, Rafael Vazquez

**Affiliations:** 1 Anesthesia, Critical Care, and Pain Medicine, Massachusetts General Hospital, Boston, USA; 2 Neuroradiology, Massachusetts General Hospital, Boston, USA

**Keywords:** regional anesthesia, minimally invasive spine, interspinous spacers, chronic and acute pain management, interventional pain medicine

## Abstract

Erector spinae plane block (ESPB) is a fascial plane block that targets the dorsal and ventral branches of the primary dorsal root ganglion. We report a case of a 76-year-old woman who presented for percutaneous posterior interspinous decompression spacer at the L3-L4 level in the setting of neurogenic claudication from severe spinal stenosis. We describe the novel performance of bilateral ESPBs under ultrasound guidance for postprocedural analgesia. Throughout the recovery period, the patient experienced sustained pain relief. ESPB may be a useful adjunct for periprocedural analgesia and recovery in patients undergoing interspinous spacer placements.

## Introduction

Erector spinae plane block (ESPB) is a fascial plane block technique that has indications for both perioperative analgesia and chronic pain management [1]. To our knowledge, it has not been applied for periprocedural analgesia of minimally invasive interspinous space decompression. Interspinous decompression is increasingly a treatment option for symptomatic lumbar spinal stenosis, with clinical evidence suggesting reduction in opioid analgesia requirements [2] and sustained five-year outcome improvements in function and quality of life [3]. In particular, interspinous spacers are finding a role as a percutaneous alternative to surgical options for elderly patients who may have significant medical co-morbidities that may preclude more open or extensive surgical procedures [4]. Currently, a paucity of literature exists to describe periprocedural strategies for analgesia and promotion of recovery in this susceptible population.

## Case presentation

A 76-year-old woman with a past medical history significant for hypertension, left bundle branch block, osteoporosis, and chronic lower back pain presented with neurogenic claudication in the setting of known spinal stenosis. She had undergone two prior augmentations of an L2 compression fracture and L4 compression fracture in the past year. However, she continued to experience persistent lower back pain consistent with spinal stenosis. Her current pharmacologic regimen included acetaminophen 500 mg twice a day (BID), gabapentin 200 mg three times a day (TID), ibuprofen 600 mg TID, and oxycodone 2.5 to 5 mg BID pro re nata (PRN). Due to concerns about compliance, she was not considered a spinal cord stimulator candidate. She was evaluated for a percutaneous posterior interspinous decompression spacer at the L3-L4 level in the setting of neurogenic claudication from severe spinal stenosis.

**Figure 1:**
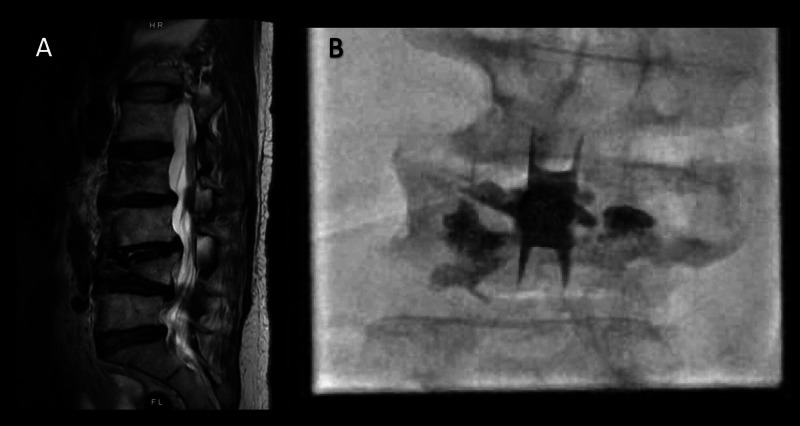
(A) Sagittal T2-weighted MRI image demonstrates vertebral compression fractures as well as multilevel discogenic and degenerative disease, worst at L3-L4. (B) Intraprocedural AP radiograph demonstrates successful placement of the superion interspinous spacer device at L3-L4. PMMA from prior augmentation is present at L4. AP, anteroposterior; PMMA, polymethyl methacrylate

The patient could not comply with sedation for the procedure. In the interventional suite, the patient was positioned prone, general anesthesia (GA) was induced, and a supraglottic airway was placed to secure the airway. A total of 50 mcg of fentanyl was used for induction. With fluoroscopic guidance, an incision was made and the supraspinous ligament was transected. A dilator was inserted at the L3-L4 space and advanced with alternating anteroposterior and lateral fluoroscopic guidance (Figure 1).

A series of dilators were used to guide the working cannula to the appropriate position dorsal to the lamina. A measuring gauge was introduced, and the space was measured. A same size device was deployed and advanced to the lamina. Procedural incisions were closed with 2-0 Vicryl sutures.

For postprocedural analgesia, bilateral ESPBs were performed under ultrasound guidance. For each side, the transverse process of L4 was identified, and a 21-gauge needle was advanced to the level of the spinous process. A total of 20 mL of 0.25% bupivacaine with 1:400,000 epinephrine was injected into each fascial plane. The patient was emerged from GA and taken to recovery. No additional intravenous narcotics were administered. Throughout the recovery period, the patient’s reported visual analog scale (VAS) was 0/10 including at arrival and at 30 minutes, 60 minutes, and 120 minutes postprocedure until discharge. The patient denied any weakness, and no motor blockade was appreciated on physical examination. Hemodynamic stability was maintained throughout the perioperative course. No additional complications were observed.

## Discussion

ESPB is a fascial plane block performed by depositing local anesthetic in the fascial plane deep to the erector spinae muscle (ESM) targeting the dorsal and ventral branches of the primary dorsal root ganglion. In recent years, ESPB has seen expanding indications in the realm of both acute and chronic pain management [5-6]. First described by Forero et al. in 2016 for two cases of severe neuropathic pain, the first from metastatic disease of the ribs and the second from malunion of rib fractures, the technique has been touted as a simple and safe analgesic technique [7].

ESPB has been described for multiple indications in the acute regional anesthesia context, including breast surgery [8], rib fractures, lumbosacral spine surgery [9], and abdominal surgery [10]. To our knowledge, ESPB has not been applied for pain management in patients undergoing percutaneous vertebral procedures including kyphoplasty, vertebroplasty, and interspinous process device (IPD) placement. The majority of percutaneous vertebral procedures are performed with moderate sedation and/or monitored anesthesia care with local anesthetic. Some patients experience significant periprocedural pain that is often compounded by chronic pain and hyperalgesic states. Pain management is frequently challenging as the majority of patients tend to be elderly with multiple co-morbidities [4]. Analgesic options are often limited to acetaminophen and opioids. Additional analgesia may be provided in this context by ESPBs, which target branches of the primary dorsal root. Analgesia is rendered to skin of the posterior abdominal wall as well as portions of the vertebrae, specifically the spinous process and lamina [1].

The ESM comprises the spinalis, longissimus thoracis, and iliocostalis muscles that function to stabilize the spine. Each bilateral set of muscles extends from the spinous to transverse process to ribs and are contiguous from the skulls to the pelvis. The goal of the ESPB is deposition of local anesthetic in a fascial plane deep to the ESM at the tip of the transverse process of the target vertebrae [5]. Both cadaveric and imaging studies have been conducted to better understand the spread and mechanism of action of ESPBs.

The original description of the ESPB was accompanied by a cadaveric investigation performed with ultrasound guidance and injection of methylene blue dye. In the first cadaver, methylene blue dye was injected between the rhomboid major muscle and ESM. Dissection was notable for longitudinal spread of dye between the erector spinae and trapezius and rhomboid major muscle with subsequent staining of the lateral branches of the dorsal rami of spinal roots. In the second cadaver, methylene blue die was injected deep to the erector spinae with visible spread to the transverse processes. Dissection was notable for spread anterior to the erector spinae with evidence of penetration deep to the intercostal muscles with extension to the ventral and dorsal rami of the spinal nerve roots [7]. In a technical report to evaluate the local anesthetic spread with ESPBs, MRI imaging was performed 45 and 90 minutes post-injection. Imaging was notable for spread of contrast both deep to the ESMs and along the paravertebral region with circumferential epidural spread through the intervertebral foramina. The mechanism suggests that the action is mediated through both the transforaminal and epidural spread [11]. Complications described have included pneumothorax [12] and unintended motor blockade [13].

A variety of IPDs have been available for decades including the Wallis Stabilization System (Zimmer, Bordeaux, France), Device for Intervertebral Assisted Motion (DIAM; Medtronic Sofamor Danek, Memphis, TN, USA), COFLEX (Paradigm Spine, New York, NY, USA), and X STOP (Medtronic Sofamor Danek). Recently, a percutaneous IPD was introduced in the United States named the SUPERION device (VertiFlex Inc., San Clemente, CA, USA). Recent data for SUPERION as stand-alone therapy have demonstrated that at five years, 84% of the patients demonstrated durable results and 75% are free of surgery, reoperation, or revision [3]. As interspinous spacers and other minimally invasive spine procedures continue to expand as viable alternatives for elderly patients, ESPBs may be a novel technique as an analgesic adjunct to promote recovery and discharge.

## Conclusions

The advantage of minimally invasive approaches continues to be amplified in patients at increased risk of surgical and anesthesia complications. Early discharge and recovery is highly desirable for this population of patients. Further studies should focus on evaluating the role and efficacy of ESPBs for enhanced periprocedural analgesia and recovery in minimally invasive spine procedures.
